# Management of donor-specific antibodies in allogeneic hematopoietic cell transplantation: A retrospective single-center case-control study

**DOI:** 10.1177/09636897261486247

**Published:** 2026-09-09

**Authors:** Lennart Harland, Semian L. Schüch, Sarah Katzenstein, Sandra Hammer, Christoph Faul, Karina Althaus, Bernhard N. Bohnert, Markus W. Löffler, Tamam Bakchoul, Claudia Lengerke, Wolfgang A. Bethge

**Affiliations:** 1 Department of Hematology, Oncology, Clinical Immunology and Rheumatology, 27203 University Hospital Tübingen, Tübingen, Germany; 2 Department of Diabetology, Endocrinology and Nephrology, 27203 University Hospital Tübingen,Tübingen, Germany; 3 Institute for Clinical and Experimental Transfusion Medicine, Medical Faculty of Tübingen, 27203 University Hospital Tübingen, Tübingen, Germany; 4 Center for Clinical Transfusion Medicine, 27203 University Hospital Tübingen, Tübingen, Germany

**Keywords:** donor-specific antibodies (DSA), engraftment, graft failure, haploidentical transplantation, HLA antibodies

## Abstract

Donor-specific antibodies (DSA) are preformed recipient antibodies directed against donor HLA antigens, most frequently observed in haploidentical or mismatched allogeneic hematopoietic cell transplantation (HCT). DSA assessed by multiplexed bead-based immunoassays at mean fluorescence intensity (MFI) values >1 000 are associated with increased risk of graft failure, graft-versus-host disease (GvHD), and mortality, supporting combined pharmacological and plasma-based depletion in absence of matching donors. We retrospectively analyzed all adult HCTs performed between 2009 and 2025 at the University Hospital Tübingen, Germany, with clinically relevant DSA (MFI >1 000). Assessments included depletion strategies, engraftment, GvHD, overall survival, and immune reconstitution. A 2:1 matched control cohort without DSA was selected based on disease, age, sex, and donor features. Fourteen cases were identified (1.2% of all transplants; 9.1% of haploidentical procedures). Depletion regimens included rituximab (n=12), bortezomib (n=10), daratumumab (n=2), intravenous immunoglobulins (n=6), plasma exchange (n=8), and immunoadsorption (n=9). MFI reduction <1 000 before HCT was achieved in 5 of 11 evaluable patients. All but two recipients engrafted timely; both graft failures occurred after single-modality depletion. Clinical outcomes did not differ significantly between DSA patients and controls. These real-world data underscore the importance of systematic DSA screening and multimodal depletion to optimize transplantation outcomes.

## Introduction

Donor-specific antibodies (DSA) are preformed anti-HLA antibodies of the recipient that are directed against HLA antigens of the donor. They play a pivotal role in the outcome of allogeneic hematopoietic cell transplantation (HCT), particularly in the context of HLA-mismatched or haploidentical transplants.^1^ The presence of DSA at the time of transplantation is a well-known risk factor for primary graft failure, as they can impede the engraftment of donor hematopoietic stem cells and adversely affect early post-transplant recovery.^2–4^ Moreover, respective anti-HLA-antibodies have been linked to an increased incidence of graft-versus-host disease (GvHD), as well as non-relapse mortality (NRM).^5^ DSA are commonly assessed using multiplexed bead-based immunoassays through mean fluorescence intensity (MFI), with MFI values >1 000 frequently considered as clinically relevant, with increasing values having been associated with an increased risk of adverse transplant outcomes.^1,6^ DSA are particularly relevant in patients with a history of sensitizing events, such as pregnancies or blood transfusions, and are more frequently detected in multiparous women and recipients of haploidentical or unrelated donor grafts.^7^ Owing to their role as barriers to engraftment, GvHD, and overall survival (OS), the necessity of routine DSA testing and appropriate depletion strategies is well established.

A variety of DSA depletion methods has been explored, although their use in conditioning regimens remains individualized. Pharmacological agents such as rituximab, bortezomib and daratumumab, as well as intravenous immunoglobulins (IVIG) have shown effectiveness in lowering measurable DSA values.^1,8–10^ In addition, extracorporeal techniques such as immunoadsorption and plasmapheresis are established for the physical removal of circulating antibodies in the transplant setting.^1^ While current recommendations favor multimodal approaches, standardized protocols for DSA depletion are not yet available, and the strategy is typically tailored to the patient’s specific risk factors and the urgency of transplantation.^4,6^

In this retrospective single center study, we evaluated all patients with DSA with MFI values >1 000, between 2009 and 2025 who underwent HCT, focusing on the applied depletion strategies, engraftment success, incidence of GvHD, and lymphocyte reconstitution.

## Patients & methods

### Patient selection and DSA definition

Patients aged 18 years or older with DSA who underwent HCT at the University Hospital Tübingen (Germany) between January 2009 and February 2025 and who subsequently received DSA depletion therapy (cases) were included in this retrospective study. Antibodies directed against donor HLA antigens were classified as DSA positive, when mean fluorescence intensity (MFI) values exceeded 1 000 using single-antigen multiplexed bead-based immunoassays.

Patient data, including demographic data, details of conditioning and DSA depletion regimens, as well as clinical data were retrieved from medical records. Engraftment and graft failure were determined according to EBMT guidelines.^11^ Additionally, donor chimerism was assessed on day +30 (±3 days) by PCR-based analysis of short tandem repeats.^12^ Donor chimerism ≥95% was defined as complete donor chimerism.

To compare clinical outcomes of our cases with an internal control group, we screened institutional databases for comparable cases with DSA negativity, defined as negative screening by multiplexed bead-based immunoassays and/or with MFI values <1 000 in single antigen multiplexed bead-based immunoassays, for whom no depletion therapy was considered. Cases and controls were frequency-matched in a 2:1 ratio, based on patient age at transplantation, sex, underlying disease, and donor features (haploidentical vs. non-haploidentical). Cases and controls were treated during the same observation period and analyzed using the identical statistical methods.

### DSA assessment

All included patients underwent DSA screening (LABScreen™ Mixed, One Lambda, Canoga Park, CA, USA) as part of routine pre-transplant evaluation. In case of a positive screening result, a Single Antigen assay was additionally performed using the LABScreen™ Single Antigen HLA Class I and II assay (One Lambda, Canoga Park, CA, USA) on the Luminex platform (Luminex Corporation, Austin, TX, USA) to further specify donor-specific antibodies. The assay is based on fluorescently labelled polystyrene microspheres coated with recombinant HLA antigens. To prevent the complement inhibition, serum samples were pretreated with EDTA^13^ and subsequently incubated with the bead mix. Present HLA antibodies bind to their respective antigens coated on the microspheres and are detectable through a subsequently added secondary phycoerythrin (PE)-labelled anti-human immunoglobulin G (anti-IgG) antibody. Human IgG-binding positive and uncoated negative latex control beads as supplied by the manufacturer (One Lambda) as well as positive and negative in-house sera were included to assess assay performance in each run.

Data acquisition and analysis was conducted using either the Luminex™ 200™ or FLEXMAP 3D™ systems with Luminex xPONENT software (Luminex Corporation, Austin, TX, USA). Luminex analyzers use a dual-laser-based excitation system for bead classification and PE detection. Importantly, the manufacturer’s adjustment of cut-off values ensured that the transition from the Luminex™ 200™ to the Luminex FLEXMAP 3D™ during the study period did not substantially affect the results.

Antibody specificity was defined by combined readouts from both lasers. The fluorescence signal from the PE label was expressed as mean fluorescence intensity (MFI). Serum reactivity was assessed by analyzing fluorescence signals for each bead with a minimum of 50 beads counted, correcting for non-specific binding to negative control beads. All data were normalized using a negative control serum.

### Antibody depletion

Pharmacological antibody depletion, including DSA, was performed using rituximab (375 mg/m^2^, intravenously), bortezomib (1.3 mg/m^2^, subcutaneously), daratumumab (1 800 mg, subcutaneously) and IVIG (1 g/kg body weight, intravenously).

Physico-immunological antibody depletion was achieved by either immunoadsorption or plasma exchange, with the indication and choice of modality determined on an individual basis. For immunoadsorption, GLOBAFFIN® (Fresenius Medical Care, Bad Homburg, Germany) was used with a target treatment volume of approximately 100–150 mL/kg body weight. In plasma exchange, the exchange volume was targeted at 0.8–1.0 times the estimated plasma volume, which was calculated using the Nadler formula.^14^

### Statistical analysis

Categorical variables were compared using the Chi-square or Fisher’s exact test, as appropriate. Continuous variables were analyzed using the two-sample *t*-test for normally distributed data (Shapiro–Wilk test) or the Mann–Whitney U test otherwise. Paired pre- and post-intervention values were compared using the Wilcoxon signed-rank test. OS was assessed using the Kaplan–Meier estimator and censored at last follow-up or up to three years. NRM, defined as death without prior disease progression, was analyzed using cumulative incidence functions accounting for competing risks. Group differences were assessed using the log-rank test (OS) and Gray’s test (NRM). All tests were two-sided with a significance level of p < 0.05. Analyses were performed using SPSS (version 30.0.0) or GraphPad Prism (version 10.6.1), while NRM was assessed using R software (version 4.6.1) utilizing the *cmprsk* package.

## Results

### DSA assessment, monitoring and depletion strategies

**Figure 1.**
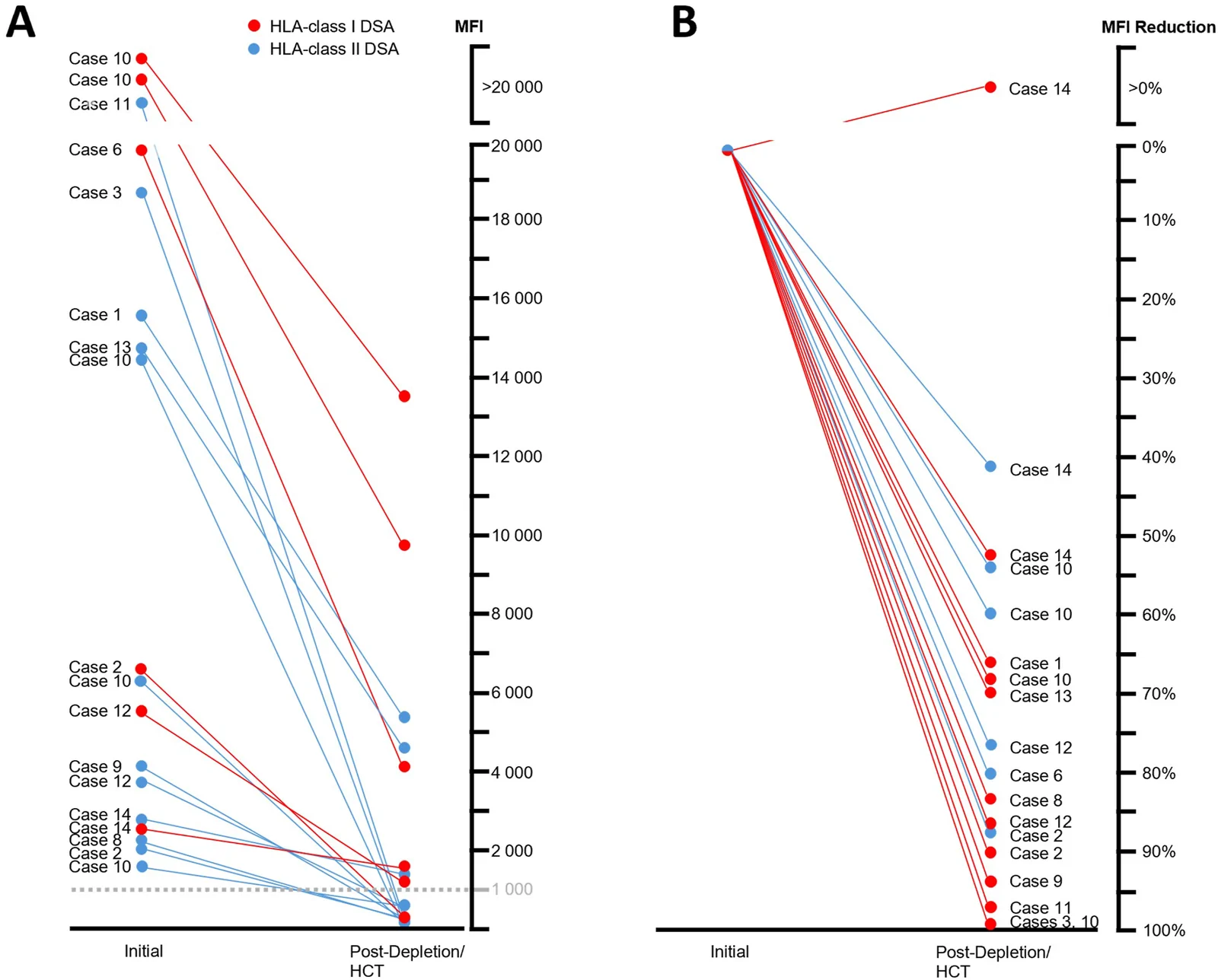
A total of fourteen cases exhibited detectable donor-specific antibodies (DSA) with a mean fluorescence intensity (MFI) >1 000; sequential MFI measurements were available for eleven of these cases. Some cases exhibited more than one DSA. All initial DSA levels prior to depletion therapy and at the time of hematopoietic cell transplantation (HCT) are presented as absolute MFI values (A) and as relative MFI reduction (B). Results are labelled according to HLA class I (red) and class II (blue) allo-antibodies.

Over a 16-year period, a total of 1 267 HCT have been conducted at our clinical center. Overall, fourteen patients (1.2%) exhibited DSA with MFI values >1 000, receiving antibody-depleting therapies (Table 1). Among them, seven patients were haploidentically transplanted, corresponding to 9.1% of all haploidentical transplantations performed during this study period. Seven patients were positive for DSA in HLA class I, three patients for DSA in HLA class II and four patients for DSA in both HLA class I and HLA class II. For eleven of these patients, longitudinal data were available providing MFI values before and after treatment. Following treatment, five cases showed MFI values <1 000 at the time of HCT. The median initial MFI was determined at 6 358 (ranging from 1 653 to 30 052), as shown in Figure 1.

**Table 1.** Characteristics of Patients with DSA undergoing HCT, including information about the underlying disease, remission status, as well as transplantation information and DSA.

Case	Sex	Age years	Disease	Remission	Regime	Donor	Donor	DSA (HLA class)	DSA (HLA locus)	MFI (initial)	MFI (at HCT)	Neutrophile recovery days	Platelet recovery days
Case 1	Female	45	AML	CR	RIC	haploident	Daughter	I	B*44:02	15 642	5 332	11	13
Case 2	Female	54	ALL	CR	RIC	haploident	Son	I, II	A*02:01 DRB1*08:04	1 981 6 477	207 763	13	9
Case 3	Female	60	AML	CR	RIC	haploident	Son	I	A*26:01	18 769	<100	GF	GF
Case 4	Female	54	ALL	CR	RIC	MMUD	unrelated	I	B*15:16	>20 000	N.a.	16	17
Case 5	Female	33	vSAA	PD	RIC	MMUD	unrelated	II	DPB1*02:01	>20 000	N.a.	15	23
Case 6	Female	64	AML	r/r	RIC	MMUD	unrelated	II	DQB1*03:03	20 600	4 039	17	12
Case 7	Female	67	AML	r/r	RIC	MMUD	unrelated	II	DPB1*02:01	>8 000	N.a.	9	17
Case 8	Male	70	PMF	PD	RIC	MMUD	unrelated	I	A*01:01	2 186	382	30	29
Case 9	Female	62	MDS	r/r	RIC	haploident	Son	I	B*44:02	4 096	237	19	25
Case 10	Female	65	AML	r/r	RIC	haploident	Son	I, II	A*01:01 B*15:01 C*03:04 DQB1*03:02 DRB1*04:01	14 486 6 238 1 653 22 941 30 052	<100 <100 535 9 180 13 723	16	20
Case 11	Female	25	AML	CR	RIC	MMUD	unrelated	I	B*39:01	22 220	725	23	28
Case 12	Female	39	AML	CR	RIC	haploident	Son	I, II	C*16:02 DQB1*03:02	3 810 5 545	512 1 347	26	14
Case 13	Female	69	AML	CR	RIC	MMUD	unrelated	I	C*01:02	14 655	4 371	26	16
Case 14	Female	56	ALL	CR	RIC	haploident	Son	I, II	A*29:01 B*07:05 DRB1*10:01	888 2 799 2 714	1 182 1 320 1 606	GF	GF

AML (acute myeloid leukemia); ALL (acute lymphocytic leukemia); CR (complete remission); DSA (donor-specific antibodies); GF (graft failure); HCT (hematopoietic cell transplantation); MDS (myelodysplastic syndrome); MMUD (mismatched unrelated donor HLA-match <10/10); PD (progressive disease); PMF (primary myelofibrosis); RIC (reduced-intensity conditioning); r/r (relapsed/refractory disease); vSAA (very severe aplastic anemia).

**Figure 2.**
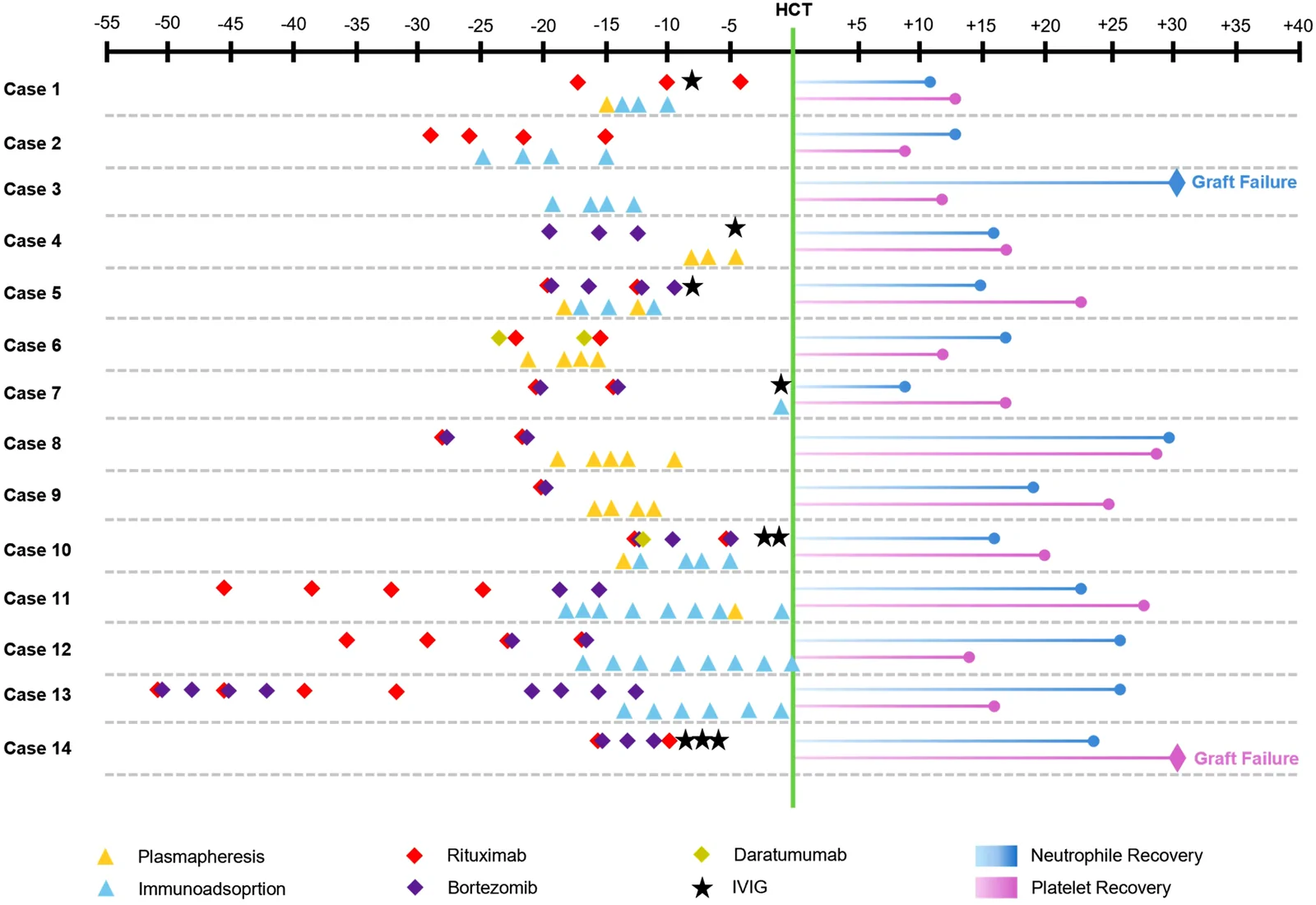
A timeline of all cases with significantly elevated donor-specific antibodies (DSA) (measured as Mean Fluorescence Index [MIF] >1 000), including information on the depletion method used and the corresponding hematologic engraftment status. Time intervals are indicated in days.

**Figure 3.**
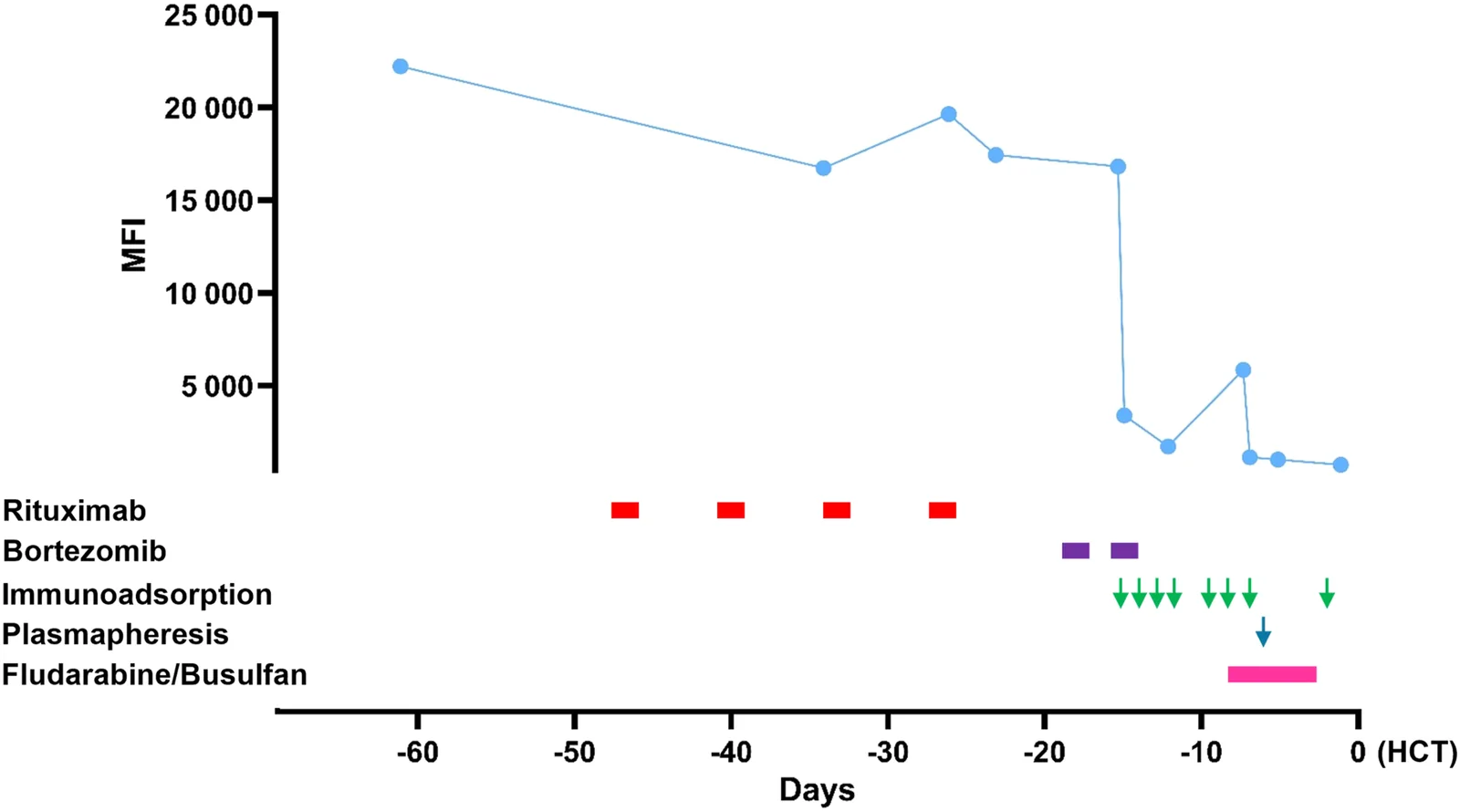
Representative mean fluorescence intensity (MFI) trends for case 11, showing a decrease during depletion treatment, with successful reduction of MFI <1 000 at the time of hematopoietic cell transplantation (HCT).

The majority of patients underwent pharmacological treatment (Figure 2). Rituximab was administered in twelve (86%), and bortezomib in ten (71%) cases, with total of three administrations per patient on average, respectively. Daratumumab was administered to two patients (14%). IVIGs were part of the depletion strategy in six (43%) cases. See also Figure 3, illustrating representative trends of DSA for case 11 in the context of depletion therapy.

**Figure 4.**
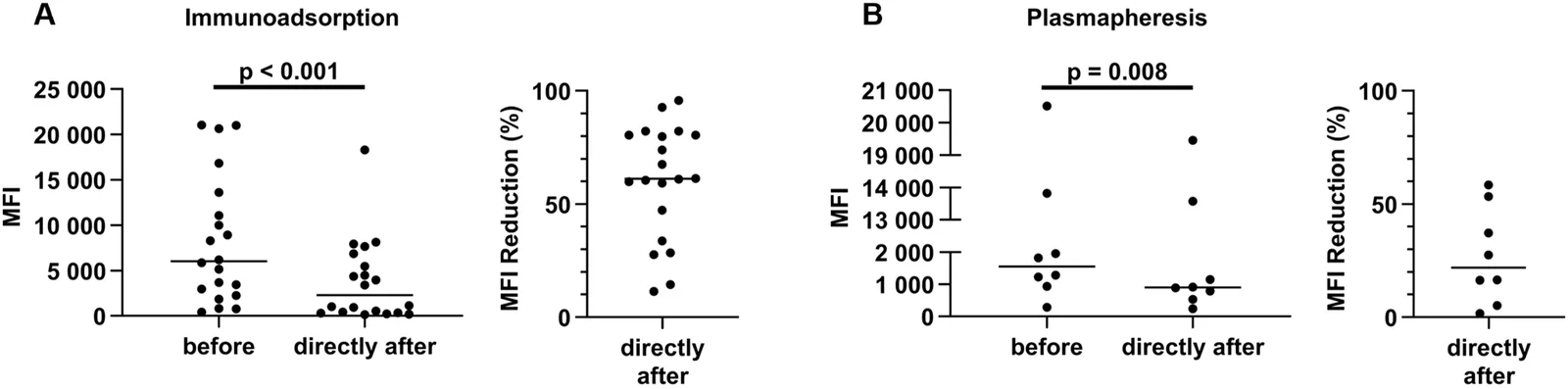
For a subset of immunoadsorption (n=20; A) and plasmapheresis (n=8; B) sessions, donor-specific antibody (DSA) levels were measured before and after treatment; however, no further analysis according to specific DSA subtypes was performed. Mean fluorescence intensity (MFI) values, as well as median for each subgroup is presented. Statistical significance was defined as p < 0.05 using Wilcoxon signed-rank test for paired, not-normally distributed values.

Immunoadsorption was conducted in nine patients (64%), with a mean of 5.4 sessions per patient. Five among those patients received immunoadsorption only, without plasma exchange. The average treatment volume was 129 mL/kg body weight (ranging from 87 to 164 mL/kg). For a subset of 20 individual immunoadsorption sessions within the whole study population, longitudinal Luminex measurements were available both before and immediately after treatment (Figure 4). Across respective sessions with paired measurements, median MFI decreased from 6 018 (IQR 2 419 to 12 986) by 2 940 immediately after treatment (p < 0.001, Wilcoxon signed-rank test). In addition, plasma exchange was performed in eight patients (57%), with a mean of 2.6 sessions per patient. Four of those patients only received plasmapheresis, but no immunoadsorptions. The average exchanged volume corresponded to 1.0 times the plasma volume (ranging from 0.6 to 1.2). For plasmapheresis, paired MFI values were available immediately after the procedure for eight sessions. The median baseline MFI was 1 555 (IQR 1 015 to 10 859), with a median reduction of 510 directly after the procedure (p = 0.008, Wilcoxon signed-rank test).

### Engraftment and clinical outcome

To compare the clinical outcomes of our patients with an internal control group, we assembled a DSA negative control group (frequency matched in a 2:1 ratio), who had not undergone depletion therapy. A comprehensive overview of patient characteristics and study endpoints is provided in Table 2.

**Table 2.** Characteristics of Patients with DSA, as well as a control group (2:1) without DSA matched for age at transplantation, gender distribution, underlying disease, as well as HLA match status.

Characteristics	DSA	Control (2:1)	
**Total**	n=14	n=28	
**Sex**	Female (n=13), Male (n=1)	Female (n=26), Male (n=2)	
**Age at HCT**, mean (median, range)	55 years (58, 25 – 70)	53 years (56, 20 – 71)	
**Disease**	AML (n=8), ALL (n=3), MDS (n=1), PMF (n=1), vSAA (n=1)	AML (n=16), ALL (n=6), MDS (n=2), PMF (n=2), vSAA (n=2)	
**DRI**	low (n=0), intermediate (n=5), high (n=5), very high (n=2)	low (n=2), intermediate (n=7), high (n=11), very high (n=4)	
**HCT-CI**	0 (n=2), 1-2 (n=6), ≥3 (n=6)	0 (n=5), 1-2 (n=11), ≥3 (n=12)	
**Follow-Up**, mean (median, range)	1768 days (1 672, 136 – 4 243)	1 963 days (1 589, 356 – 4 011)	
**Transplantation**			
**Conditioning regime**	RIC (n=14), MAC (n=0)	RIC (n=26), MAC (n=2)	
**Donor type**	haploident (n=7), non-haploident (MMUD n=7, MUD n=0)	haploident (n=14), non-haploident (MMUD n=8, MUD n=6)	
**Source**	PBSC (n=13), BM (n=1)	PBSC (n=26), BM (n=2)	
**CD34+** **cell count/kg**, mean (range)	7.1 x 10^6^ (2.8 – 17 x 10^6^)	7.9 x 10^6^ (1.6 – 17.4 x 10^6^)	
**Immunosuppression**	MMF (n=11), postCy (n=7), ATG (n=6), Tacrolimus (n=6), CSA (n=5), MTX (n=2)	MMF (n=19), postCy (n=10), ATG (n=17), Tacrolimus (n=16), CSA (n=8), MTX (n=8)	
**Engraftment**			
**Primary Graft Failure**	n=2 (excluded from Platelet-/Neutrophile-Kinetics)	n=1 (excluded from Platelet-/Neutrophile-Kinetics)	
**Neutrophile Recovery**, mean (median, range)	18.4 days (17, 9 - 30)	20.7 days (20, 10 - 33)	
**Platelet Recovery**, mean (median, range)	18.6 days (17, 9 - 29)	19.5 days (18, 10 – 40)	
**Chimerism (Day 30)**	Complete Chimerism (n=14)	Complete Chimerism (n=28)	
**Outcome**			
**Non-Relapse-Mortality (Day 100)**	7% (95% CI 0.0 – 29%)	4% (95% CI 0 – 16%)	
**Non-Relapse-Mortality (1 Year)**	14% (95% CI 2 – 38%)	11% (95% CI 3 – 25%)	
**Overall-Survival (1 Year)**	71% (95% CI 48 – 95%)	82% (95% CI 68 – 96%)	
**Overall-Survival (3 Years)**	64% (95% CI 39 – 89%)	75% (95% CI 59 – 91%)	
**GvHD**			
**Acute GvHD**, Grades	I (n=2), II (n=6), III (n=0), IV (n=2)	I (n=8), II (n=9), III (n=3), IV (n=0)	
**Acute GvHD**, manifestation	Skin (n=5), Gastrointestinal (n=3), Liver (n=1), CNS (n=1)	Skin (n=19), Gastrointestinal (n=6), Liver (n=1)	
**Chronic GvHD**, severity	Mild (n=3), Moderate (n=1), Severe (n=2)	Mild (n=8), Moderate (n=3), Severe (n=2)	
**Chronic GvHD**, manifestation	Skin (n=6), Gastrointestinal (n=1), Liver (n=1), CNS (n=1)	Skin (n=11), Gastrointestinal (n=6), Liver (n=1)	
**Lymphocyte Count (Day 100)**			
**Total Lymphocytes**, mean (median, range)	1 203/µL (1 260, 253 – 2 493)	1 117/µL (1 066, 60 – 3 120)	
**CD4**^**+**^ **Lymphocytes**, mean (median, range)	207/µL (188, 28 – 607)	164/µL (147, 3 – 504)	
**CD8**^**+**^ **Lymphocytes**, mean (median, range)	642/µL (460, 15 – 1 944)	500/µL (478, 2 – 1 463)	

AML (acute myeloid leukemia); ALL (acute lymphocytic leukemia); ATG (Antithymocytic Globulin); BM (bone marrow); CSA (Ciclosporin); DRI (Disease Risk Index); DSA (donor-specific antibodies); GvHD (Graft-versus-Host Disease); HCT (hematopoietic cell transplantation); HCT-CI (Hematopoietic Cell Transplantation-specific Comorbidity Index); MAC (myeloablative conditioning); MDS (myelodysplastic syndrome); MMF (Mycophenolate Mofetil); MMUD (mismatched unrelated donor HLA-match <10/10); MUD (matched unrelated donor, HLA-match 10/10); MTX (Methotrexate); PBSC (peripheral blood stem cells); PMF (primary myelofibrosis); postCy (post-transplant cyclophosphamide); RIC (reduced-intensity conditioning); vSAA (very severe aplastic anemia).

The majority of patients exhibited successful engraftment following HCT, without statistically significant differences between the two groups concerning neutrophil granulocyte engraftment (p = 0.303, unpaired *t*-test), platelet engraftment (p = 0.735, unpaired *t*-test), as well as lymphocyte recovery at day 100 (p = 0.784, unpaired *t*-test). Three patients were excluded from engraftment kinetics due to primary graft failure; all of them underwent haploidentical transplantation. One patient in each group experienced delayed engraftment, engrafting ultimately following G-CSF stimulation and optimization of immunosuppression. Additionally, case 14 among the DSA cohort showed incomplete platelet engraftment, necessitating a CD34+ boost on day 75 post HCT, due to failure of achieving transfusion independence. Notably, all cases demonstrated complete chimerism around day 30 post HCT. While DSA in case 3 could be successfully depleted, case 14 retained three different DSA with MFI values >1 000. Interestingly, those two patients were the only cases without a combined depletion strategy of pharmacological intervention and extracorporeal elimination: case 3 was only treated with immunoadsorption and case 14 only received two doses of rituximab, three doses of bortezomib and IVIGs.

Both groups resulted well balanced with respect to the Hematopoietic Cell Transplantation Comorbidity Index (HCT-CI) and Disease Risk Index (DRI) and demonstrated comparable outcomes in terms of OS and NRM. The 1-year OS was 71% (95% CI 48–95%) in the DSA group and 82% (95% CI 68–96%) in the control group, while the 3-year OS was 64% (95% CI 39–89%) and 75% (95% CI 59–91%), respectively. The cumulative incidence of NRM at day 100 was 7% in the DSA group and 4% in the control group, increasing to 14% and 11% at 1 year, respectively. However, these differences were not statistically significant (Gray’s test, p = 0.71).

By the end of follow-up, a total of five deaths had occurred in the DSA group. Cases 9 and 13 died due to disease progression, while case 6 succumbed to aspergillosis. Two additional deaths occurred in patients with severe neuroinflammatory syndromes clinically interpreted as CNS manifestations of GvHD. Case 3 developed rapidly progressive encephalitis followed by tetraparesis in the setting of grade IV acute GvHD, whereas case 8 presented several months after HCT with cerebellar ataxia and dysarthria, consistent with chronic CNS-GvHD. Although these neurological complications were rare, both proved fatal.

Acute and chronic GvHD occurred at similar frequencies in both groups. Acute GvHD grade ≤ II was observed in 57% of patients in the DSA group and 61% in the control group, predominantly affecting the skin and gastrointestinal tract. Severe acute GvHD occurred in two patients in the DSA group and three patients in the control group. Neither the incidence of acute GvHD (p = 0.626, Mann–Whitney U test) nor chronic GvHD (p = 0.841, Mann–Whitney U test) differed significantly between the groups.

## Discussion

At our center, approximately 1% of all allogeneic transplant recipients required antibody depletion for pre-formed DSA >1 000 MFI, while approximately 10% of haploidentical transplant recipients exhibited DSA above this threshold. As DSA screening is routinely performed whenever an HLA mismatch is anticipated, the prevalence in the haploidentical cohort is considered comprehensive. The overall incidence may, however, represent a slight underestimation because isolated patients with low-level DSA not requiring antibody depletion may not have been captured retrospectively. Nevertheless, the observed incidence is consistent with previous reports, particularly regarding the higher prevalence among parous women.^15–17^ Assessing respective constellations between donors and recipients constitutes a challenge for pretransplant coordination, as does the characterization of DSA. Multiple technical limitations exist regarding the interpretation of MFI values in the context of DSA, since respective values are inherently semi-quantitative and subject to inter-laboratory variability.^18–20^ Additionally, antibody characteristics display substantial heterogeneity, particularly in terms of biological function, including their complement-binding capacity, which may critically determine their damaging potential directed towards the allograft.^21^ This heterogeneity precludes the determination of robust clinical cut-off values. Consequently, when aberrant findings emerge during screening, supplementary assays, such as C1q reactivity may support the accurate estimation of the associated immunological risk from DSA in terms of graft injury.^6^ Morevover, false-positive reactions have been described in the literature, involving reactions to denatured HLAs in single antigen multiplexed bead-based immunoassays, resulting in typical patterns of positivity for both HLA-class I and HLA class II.^22,23^ While we are generally aware of such phenomena, in absence of a systematic vetting of DSAs using a cell-based assay, respective artifacts cannot be completely ruled out and constitute a further limitation of this work.

Recent evidence suggests that the feasibility of DSA depletion should be assessed using a multimodal approach integrating bead-based testing with functional cell-based assays and antibody titration.^24^ This strategy may distinguish permissive DSA, amenable to desensitization, from clinically significant DSA, typically characterized by positive CDC crossmatches or high antibody titers and associated with a substantial risk of graft failure despite desensitization. Serial bead-based DSA measurements remain valuable for longitudinal monitoring of antibody responses during desensitization, as illustrated by the variable treatment responses observed among individual DSA in case 14.

In this cohort, most patients experienced a significant reduction of DSA values; by the time of stem cell infusion, 45% of patients exhibited MFI values <1 000. Due to the heterogeneous biological characteristics of DSA and individualized patient management, no single depletion strategy can be definitively recommended. In our cohort, the two patients only being treated with either therapeutic apheresis or pharmacological approaches developed primary graft failure (cases 3 & 14). As exemplified by Case 11, we follow a strategy combining early pharmacological intervention with the initiation of apheresis procedures immediately prior to the start of conditioning. Throughout all treatment phases, DSA are supposed to be monitored regularly, allowing adjustment of the intensity of apheresis or modification of the apheresis modality according to clinical urgency. Current guidelines support this multimodal approach; however, bortezomib is discouraged under the current ASTCT consensus recommendations, since prospective studies have demonstrated only limited efficacy.^6,25^

Our results indicate that immunoadsorption can achieve rapid initial antibody reduction, although subsequent shifts in antibody serum levels may occur shortly thereafter. The apparently lower effectiveness of immunoadsorption in some cases may largely be attributable to lower initial MFI values; patients with low MFI values from the beginning were often treated exclusively with plasma separation (i.e. cases 8 & 9). When only low amounts of DSA are present prior to treatment, antibody redistribution may prevent a noticeable decrease, while observed changes likely reflect true antibody elimination. In contrast, non-regenerable immunoadsorption systems may become saturated with DSA, leading to only minimal measurable pre–post treatment differences, whereas regenerable systems, as used in our cohort, are less prone to this limitation. Prior studies suggest that some antibodies may form complexes or prevail in protein-bound states, implying that plasma separation might represent a more effective strategy for selected cases.^26,27^ Importantly, our data does not support a preference of one over the other modality. Our cohort is too small for any definitive conclusions, and an individualized choice of apheresis method based on the number and amount of DSA as well as the patient’s clinical general condition, such as other factors including coagulation status is likely optimal. Serial monitoring of DSA remains essential to guide modality selection and to assess treatment efficacy.

Due to the small size as well as the heterogeneity of the assessed cohorts in terms of age, underlying disease, and transplantation modality, definitive conclusions regarding clinical outcomes are inadmissible. Isolated reports have described patients with DSA who did not undergo depletion procedures; however, at our center, the detection of highly elevated DSA mostly prompted depletion interventions. Consequently, a matched control group without DSA was selected at a 2:1 ratio, with similar characteristics, to evaluate the aforementioned clinical parameters. In our cohort, no statistically significant differences regarding engraftment, NRM, GvHD incidence and severity, or immune reconstitution were observed between the two groups. Ciurea et al. reported that the cumulative incidence of graft failure was 2.7% in patients without DSA compared to 37% in those with DSA.^28^ According to a more recent systematic review and meta-analysis, the presence of DSA before HCT is associated with a 7.47-fold increased risk of primary graft failure compared to patients lacking respective antibodies.^2^

Interestingly, cases 3 and 8 developed severe neurological disorders during follow-up that were clinically interpreted as CNS-GvHD and resulted in fatal outcomes. As histopathological confirmation was unavailable and CNS-GvHD represents an exceptionally rare complication, these observations should be interpreted with caution.^29^ To our knowledge, an association between preformed DSAs and CNS-GvHD has not been reported previously, and our observations do not allow any conclusions regarding causality, particularly in light of the marked decline in DSA levels following depletion therapy.

In summary, our findings underscore the importance of routine pre-transplant DSA screening and, when indicated, the implementation of appropriate DSA-depletion or desensitization strategies to optimize transplantation outcomes. Our real-world data illustrate the use of diverse desensitization approaches, encompassing both pharmacological suppression of antibody production and plasma-based depletion procedures. A multimodal approach, combined with close monitoring during therapy, appears to be the most prudent strategy. Further research is warranted to elucidate the underlying mechanisms by which DSA affect clinical outcomes and to refine management protocols for affected patients.

## Data Availability

The datasets generated and/or analyzed during the current study are available from the corresponding author on reasonable request.*

## References

[1] Ciurea SO Cao K Fernandez-Vina M , et al. The European Society for Blood and Marrow Transplantation (EBMT) Consensus Guidelines for the Detection and Treatment of Donor-specific Anti-HLA Antibodies (DSA) in Haploidentical Hematopoietic Cell Transplantation. Bone Marrow Transplant 2018; 53(5): 521–534. 10.1038/s41409-017-0062-8 29335625 PMC7232774

[2] Xie Y Parekh J Tang Z , et al. Donor-Specific Antibodies and Primary Graft Failure in Allogeneic Hematopoietic Stem Cell Transplantation: A Systematic Review and Meta-Analysis. Transplant Cell Ther 2021; 27(8): 687e1. 10.1016/j.jtct.2021.04.030 33989833

[3] Huang Y Luo C Wu G , et al. Effects of donor-specific antibodies on engraftment and long-term survival after allogeneic hematopoietic stem cell transplantation-A systematic review and meta-analysis. Bone Marrow Transplant 2023; 58(5): 544–551. 10.1038/s41409-023-01932-6 36782066

[4] Zhou Y Chen YL Huang XY , et al. Desensitization Strategies for Donor-Specific Antibodies in HLA-Mismatched Stem Cell Transplantation Recipients: What We Know and What We Do Not Know. Oncol Ther 2024; 12(3): 375–394. 10.1007/s40487-024-00283-6 38879734 PMC11333671

[5] Chang YJ Zhao XY Xu LP , et al. Donor-specific anti-human leukocyte antigen antibodies were associated with primary graft failure after unmanipulated haploidentical blood and marrow transplantation: a prospective study with randomly assigned training and validation sets. J Hematol Oncol 2015; 8: 84. 10.1186/s13045-015-0182-9 26156584 PMC4496923

[6] Kongtim P Vittayawacharin P Zou J , et al. ASTCT Consensus Recommendations on Testing and Treatment of Patients with Donor-specific Anti-HLA Antibodies. Transplant Cell Ther 2024; 30(12): 1139–1154. 10.1016/j.jtct.2024.09.005 39260570 PMC12637036

[7] Fingrut WB Davis E Archer A , et al. Gender disparities in allograft access due to HLA-sensitization in multiparous women. Blood Adv 2024; 8(2): 403–406. 10.1182/bloodadvances.2023011893 38029385 PMC10820334

[8] Ciurea SO Al Malki MM Kongtim P , et al. Treatment of allosensitized patients receiving allogeneic transplantation. Blood Adv 2021; 5(20): 4031–4043. 10.1182/bloodadvances.2021004862 34474478 PMC8945639

[9] Zheng X Yan H Han D , et al. Successful desensitization of high level donor-specific anti-HLA antibody in patients with hematological diseases receiving haploidentical allografts. Ann Hematol 2022; 101(8): 1777–1783. 10.1007/s00277-022-04844-5 35726105

[10] Zhu J Wang Q Liu Y , et al. High-Dose immunoglobulin Intervention as an effective and simple strategy for donor specific Anti-HLA antibody desensitization in haploidentical transplant. Int Immunopharmacol 2023; 120: 110299. 10.1016/j.intimp.2023.110299 37201405

[11] Sureda A Carpenter PA Bacigalupo A , et al. Harmonizing definitions for hematopoietic recovery, graft rejection, graft failure, poor graft function, and donor chimerism in allogeneic hematopoietic cell transplantation: a report on behalf of the EBMT, ASTCT, CIBMTR, and APBMT. Bone Marrow Transplant 2024; 59(6): 832–837. 10.1038/s41409-024-02251-0 38443706 PMC11161398

[12] Bader P Beck J Frey A , et al. Serial and quantitative analysis of mixed hematopoietic chimerism by PCR in patients with acute leukemias allows the prediction of relapse after allogeneic BMT. Bone Marrow Transpl 1998; 21(5): 487–495. 10.1038/sj.bmt.1701119 9535041

[13] Schnaidt M Weinstock C Jurisic M , et al. HLA antibody specification using single-antigen beads--a technical solution for the prozone effect. Transplantation 2011; 92(5): 510–515. 10.1097/TP.0b013e31822872dd 21869744

[14] Nadler SB Hidalgo JH Bloch T . Prediction of blood volume in normal human adults. Surgery 1962; 51(2): 224–232. 21936146

[15] Altareb M Al-Awwami M Alfraih F , et al. Incidence and significance of donor-specific antibodies in haploidentical stem cell transplantation. Bone Marrow Transplant 2023; 58(6): 680–686. 10.1038/s41409-023-01950-4 36959370

[16] La Rocca U Perrone MP Piciocchi A , et al. Donor specific anti-HLA antibodies in hematopoietic stem cell transplantation. Single Center prospective evaluation and desensitization strategies employed. Blood Transfus 2024; 22(2): 157–165. 10.2450/BloodTransfus.464 37847211 PMC10920073

[17] Carter M Taniguchi M Yang D , et al. Donor-specific HLA antibodies associate with chronic graft-versus-host disease in haploidentical hematopoietic stem cell transplantation with post-transplant cyclophosphamide. Bone Marrow Transplant 2022; 57(1): 134–136. 10.1038/s41409-021-01494-5 34635797 PMC8860343

[18] Sullivan HC Gebel HM Bray RA . Understanding solid-phase HLA antibody assays and the value of MFI. Hum Immunol 2017; 78(7-8): 471–480. 10.1016/j.humimm.2017.05.007 28576578 PMC8663030

[19] Sullivan HC Liwski RS Bray RA , et al. The Road to HLA Antibody Evaluation: Do Not Rely on MFI. Am J Transplant 2017; 17(6): 1455–1461. 10.1111/ajt.14229 28199773 PMC8663029

[20] McCaughan J Xu Q Tinckam K . Detecting donor-specific antibodies: the importance of sorting the wheat from the chaff. Hepatobiliary Surg Nutr 2019; 8(1): 37–52. 10.21037/hbsn.2019.01.01 30881964 PMC6383008

[21] Ciurea SO Thall PF Milton DR , et al. Complement-Binding Donor-Specific Anti-HLA Antibodies and Risk of Primary Graft Failure in Hematopoietic Stem Cell Transplantation. Biol Blood Marrow Transplant 2015; 21(8): 1392–1398. 10.1016/j.bbmt.2015.05.001 25985919 PMC4506716

[22] Grenzi PC de Marco R Silva RZ , et al. Antibodies against denatured HLA class II molecules detected in luminex-single antigen assay. Hum Immunol 2013; 74(10): 1300–1303. 10.1016/j.humimm.2013.06.035 23831256

[23] Gutierrez-Larranaga M Riesco L Guiral S , et al. Detection of antibodies to denatured human leucocyte antigen molecules by single antigen Luminex. HLA 2021; 97(1): 52–59. 10.1111/tan.14098 33040479

[24] Gocke CB Zahurak M Sinanidis I , et al. Personalized Desensitization for Donor-Specific Anti-HLA Antibodies in Allogeneic Blood or Marrow Transplantation. Transpl Cell Ther 2026; 32(5): 561–561.e16. 10.1016/j.jtct.2025.11.009 41207383

[25] Choe H Gergis U Hsu J , et al. Bortezomib and Immune Globulin Have Limited Effects on Donor-Specific HLA Antibodies in Haploidentical Cord Blood Stem Cell Transplantation: Detrimental Effect of Persistent Haploidentical Donor-Specific HLA Antibodies. Biol Blood Marrow Transplant 2019; 25(2): e60–e64. 10.1016/j.bbmt.2018.10.018 30661542

[26] Hug MN Keller S Marty T , et al. HLA antibody affinity determination: From HLA-specific monoclonal antibodies to donor HLA specific antibodies (DSA) in patient serum. HLA 2023; 102(3): 278–300. 10.1111/tan.15047 37191252

[27] Murali TM Gu Y Minhat RA , et al. Antigen-antibody complex density and antibody-induced HLA protein unfolding influence Fc-mediated antibody effector function. Front Immunol 2024; 15: 1438285. 10.3389/fimmu.2024.1438285 39744638 PMC11688311

[28] Ciurea SO Thall PF Wang X , et al. Donor-specific anti-HLA Abs and graft failure in matched unrelated donor hematopoietic stem cell transplantation. Blood 2011; 118(22): 5957–5964. 10.1182/blood-2011-06-362111 21967975 PMC3761379

[29] Lambert N Forte F El Moussaoui M , et al. CNS manifestations in acute and chronic graft-versus-host disease. Brain 2025; 148(4): 1122–1133. 10.1093/brain/awae340 39442000 PMC11967822

